# Active Component Design of Amorphous SnP_x_/SnS_x_ and Interfacial Bonding Engineering in N/P/S‐Doped Hard Carbon for High‐Rate Sodium‐Ion Hybrid Capacitors

**DOI:** 10.1002/advs.202506532

**Published:** 2025-05-28

**Authors:** Ziyang Jia, Yichen Duan, Xi Chen, Zewen Sun, Lili Liu, Lijun Fu, Yuhui Chen, Faxing Wang, Tao Wang, Yuping Wu

**Affiliations:** ^1^ School of Energy Science and Engineering Nanjing Tech University Nanjing Jiangsu 211816 China; ^2^ Confucius Energy Storage Lab School of Energy and Environment & Z Energy Storage Center Southeast University Nanjing 211189 China

**Keywords:** amorphous engineering, anode, hard carbon, hybrid capacitor, sodium ion

## Abstract

Sodium‐ion hybrid capacitors （SIC）face critical challenges from the kinetic mismatch and cycling life imbalance between battery‐type anodes and capacitive cathodes. A slope‐dominant N/P/S‐doped hard carbon anode (Sn0.1@NSPC) with nearly plateau‐free sloping charge–discharge curves, embedded with amorphous SnP_x_/SnS_x_ composites, is developed. This unique design delivers a high reversible capacity of 412.8 mAh g⁻¹ at 0.05 A g⁻¹ while retaining 180.7 mAh g⁻¹ at 10 A g⁻¹, coupled with 90% capacity retention over 10 000 cycles. The amorphous SnP_x_/SnS_x_ enables isotropic Na⁺ diffusion and volume expansion suppression, while interfacial Sn─P/Sn─S bonding activates the redox potential of P/S for sodium storage through reversible Na₃P/Na₂S formation. Density functional theory calculations demonstrate that Sn doping enhances electronic states near the Fermi level and reduces sodium‐ion diffusion barriers, improving conductivity and ion transport. Pseudocapacitive‐dominated kinetics with reduced charge transfer resistance are achieved, synergizing with alloying/conversion reactions. In SIC paired with activated carbon, the system exhibits an energy density of 360 Wh kg⁻¹ (anode‐mass‐based), a power density of 38 kW kg⁻¹, and 91% capacity retention after 3000 cycles. This work establishes a universal heterostructure design via amorphous engineering and interfacial coupling, addressing trade‐offs between high capacity, rapid kinetics, and long‐term cycling stability in advanced SIC.

## Graphical abstract

The incorporation of Sn in the hard carbon doped with nonmetallic heteroatoms enhances the number of active sites and activates the sodium storage capacity of P and S, while the carbon‐based heterostructure improves electronic conductivity and stabilizes the structure during cycling.

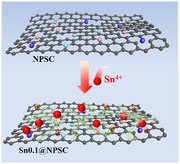

## 1 Introduction

Sodium‐ion hybrid capacitors (SICs) have emerged as promising alternatives to lithium‐ion hybrid capacitors, combining exceptional energy density from battery‐type electrodes with impressive power density from capacitor‐type electrodes. Additionally, SICs benefit from lower costs, improved safety, and broader operating temperature ranges due to the favorable electrochemical properties of sodium.^[^ ^1^, ^2^, ^3^ ^]^ However, SICs face a significant challenge in the kinetic mismatch between battery‐type materials, which rely on Faradaic reactions, and capacitor‐type materials, which operate based on adsorption–desorption mechanisms.^[^ ^4^, ^5^, ^6^ ^]^ Furthermore, the large radius of sodium ions exacerbates the challenge by causing significant volume changes and structural instability in battery‐type electrode materials during sodiation and desodiation. This leads to an imbalance in the cycling life of the cathode and the anode.^[^ ^7^, ^8^ ^]^ To overcome these challenges, the development of anodes with high reversible capacity, fast kinetics, and excellent cycling stability has become crucial for advancing SIC technology.^[^ ^9^ ^]^

Hard carbon is widely regarded as a promising anode material for commercial sodium‐ion batteries.^[^ ^10^, ^11^ ^]^ However, the coexistence of high‐potential sloping capacity (>0.1 V vs Na/Na⁺) and low‐potential plateau capacity (0.1–0 V vs Na/Na⁺) poses significant challenges to superior rate performance.^[^ ^12^ ^]^ This issue arises primarily from the proximity of the low‐potential plateau to the deposition potential of metallic sodium, as well as the diffusion‐controlled kinetics associated with the plateau capacity.^[^ ^13^ ^]^ Current research efforts focus on optimizing the parameters of closed pores linked to plateau capacity.^[^ ^14^, ^15^ ^]^ While these optimizations improve the rate performance of hard carbon, the presence of low‐potential plateau capacity remains a limiting factor. Thus, one potential solution is to reduce or eliminate the low‐potential plateau by integrating high‐capacity anode materials that exhibit sloping potential curves. This strategy can mitigate the impact of plateau capacity on rate performance without sacrificing overall capacity. Composite anode materials with sloping potential curves, such as graphene‐Nb₂O₅ ^[^ ^16^ ^]^ and black phosphorus‐graphite,^[^ ^17^ ^]^ have demonstrated excellent kinetics and rate performance. Moreover, the built‐in electric field within carbon‐based heterostructures with sloping potential curves enhances rapid ion and electron transport, further improving rate performance.^[^ ^18^, ^19^ ^]^ These findings highlight the importance of designing anode materials using slope‐dominant hard carbon‐based heterostructures to address the kinetic challenges of sodium‐ion hybrid capacitors.

Alloy‐type anode materials (e.g., P, Sn, Sb, Bi) are highly attractive due to their superior theoretical capacities and favorable operating potential plateaus. Among these, Sn‐based materials exhibit high theoretical capacities (SnP:1209 mAh g⁻¹; SnS₂:1137 mAh g⁻¹; SnS:1022 mAh g⁻¹) and appropriate working potentials (0.1–1.0 V vs Na⁺/Na). Their multistep reaction pathways, characterized by a synergistic conversion–alloying mechanism, can significantly enhance sodium storage activity while suppressing the formation of sodium dendrites, thereby ensuring electrochemical safety.^[^ ^20^, ^21^, ^22^ ^]^ However, their practical implementation is severely hindered by electrode pulverization caused by anisotropic crystal expansion during Na⁺ storage.^[^ ^23^, ^24^, ^25^ ^]^ Amorphous engineering addresses this challenge through isotropic strain distribution in disordered structures, which homogenizes mechanical stresses during sodiation–desodiation and effectively mitigates structural collapse.^[^ ^26^, ^27^ ^]^ Concurrently, the open network architecture of amorphous alloys facilitates low‐tortuosity ion transport pathways, achieving enhanced effective diffusion coefficients.^[^ ^28^, ^29^, ^30^ ^]^ Moreover, defect‐induced localized states activate multi‐redox reactions, providing abundant electroactive sites for Na⁺ storage. Consequently, amorphous engineering is particularly suited for high‐capacity alloy anodes, as it synergistically improves structural stability and accommodates intrinsic volume fluctuations inherent to Na⁺ insertion/extraction processes. It is worth noting that amorphous materials suffer from inherently low electronic conductivity due to their long‐range disordered structure; consequently, strategies such as constructing heterogeneous interfaces or introducing gradient conducting carriers are commonly employed to optimize interfacial charge transfer kinetics.^[^ ^31^, ^32^, ^33^ ^]^

This study developed a synthesis strategy utilizing the strong chelating capability of phytic acid and the templating properties of polyaniline to construct a novel anode material comprising amorphous SnP_x_/SnS_x_ composites (Sn0.1@NPSC) encapsulated within a slope‐dominant hard carbon matrix. The heteroatom (N/P/S)‐enriched hard carbon framework (NPSC) enables uniform dispersion and high loading of Sn species through chemical coordination, while effectively suppressing volume variations of the amorphous SnP_x_/SnS_x_ during cycling. The amorphous SnP_x_/SnS_x_ phase mitigates sodiation‐induced stress via isotropic Na⁺ diffusion channels and stress‐buffering interfaces, whereas the heteroatom‐modified carbon matrix activates multi‐step redox reactions (reversible Na₃P/Na₂S formation) and optimizes charge distribution through interfacial Sn─P/Sn─S bonding. The synergistic carbon‐based heterointerface imparts pseudocapacitance‐dominated kinetics (88% contribution at 1 mV s⁻¹) to the material, effectively mitigating the kinetic limitations of conventional conversion–alloying mechanisms. Coupled with a highly conductive carbon network and hierarchically porous architecture, the Sn0.1@NPSC anode delivers a reversible capacity of 412.8 mAh g⁻¹ at 0.05 A g⁻¹, maintains 180.7 mAh g⁻¹ at 10 A g⁻¹, and achieves 90% capacity retention over 10 000 cycles through strain‐adaptive mechanisms at the amorphous carbon interface. Additionally, the assembled sodium‐ion hybrid capacitor (SIC) achieves a remarkable energy density of 360 Wh kg⁻¹ based on anode mass and an extended cycle life of 3000 cycles. This work proposes an effective strategy for designing slope‐dominant hard carbon‐based composite electrodes with enhanced rate capability and cycling stability, offering a valuable approach to mitigate the kinetic and cycle life mismatches between the cathode and anode in hybrid capacitors.

## 2 Results and Discussion

**Figure 1:**
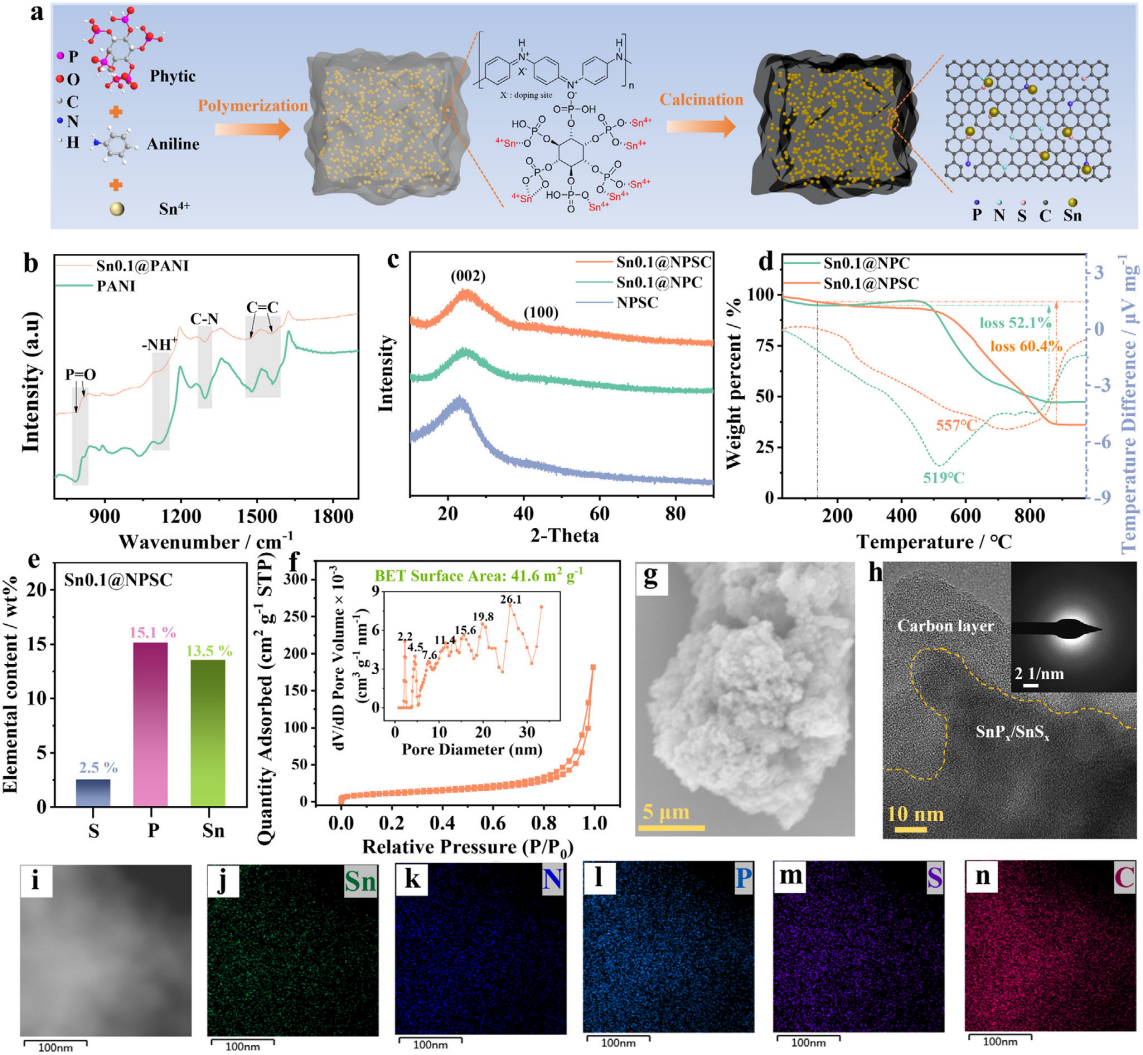
The physical‐chemical characterization of Sn0.1@NPSC. a) The synthesis process of Sn0.1@NPSC. b) FTIR, c) XRD, d) TG of the different samples. e) ICP‐OES, f) N_2_ adsorption–desorption isotherms and pore distributions (inset), g) SEM, (h) HRTEM and SAED (inset), and (i‐n) selected area and the corresponding mapping of Sn0.1@NPSC.

The preparation of the amorphous SnP_x_/SnS_x_ composite encapsulated in slope‐dominant hard carbon (Sn0.1@NPSC) is illustrated in **Figure** **1**a. Polyaniline (PANI), a widely used precursor for hard carbon due to its high carbon yield, was selected as the initial carbon source.^[^ ^34^, ^35^ ^]^ Phytic acid was chosen as a protonating dopant in the aniline polymerization process, to leverage its phosphorus‐rich structure and strong chelating ability with Sn ions.^[^ ^36^, ^37^ ^]^ Sulfur was introduced during the carbonization stage to create additional active sites and facilitate the formation of heterogeneous interfaces.^[^ ^38^, ^39^ ^]^

Both PANI and Sn0.1@PANI exhibit characteristic peaks corresponding to quinonoid C═C, benzenoid C═C, C─N, ─NH⁺, and P═O (Figure 1b). These peaks indicate that the incorporation of Sn⁴⁺ did not alter the structure of the phytic acid‐doped PANI.^[^ ^40^ ^]^ To evaluate the effect of P, S, and Sn on the slope‐dominant hard carbon structure, three anode materials were prepared: P and S co‐doped slope‐dominant hard carbon (NPSC), P and Sn co‐doped slope‐dominant hard carbon (Sn0.1@NPC), and P, S, and Sn co‐doped slope‐dominant hard carbon (Sn0.1@NPSC).

X‐ray diffraction (XRD) patterns confirm that all three materials exhibit amorphous structures, which verifies that Sn in Sn0.1@NPC and Sn0.1@NPSC is present as an amorphous phase (Figure 1c). Thermogravimetric analysis (TG analysis reveals that 52.1% and 60.4% of the mass is lost during the conversion of Sn0.1@NPC and Sn0.1@NPSC to SnO_2_, respectively. This mass loss underscores the carbon‐dominated nature of these composites (Figure 1d). Additionally, inductively coupled plasma optical emission spectrometry (ICP‐OES) (Figure 1e) reveals that Sn0.1@NPSC contains 2.5 wt.% sulfur, 15.1 wt.% phosphorus, and 13.5 wt.% tin. Furthermore, Brunauer‐Emmett‐Teller BET analysis (Figure 1f) shows that Sn0.1@NPSC has a specific surface area of 41.6 m^2^ g⁻¹ with abundant mesopores, which contribute to active site availability and ion transport during cycling. Scanning electron microscopy (SEM) and Transmission electron microscopy (TEM) images demonstrate that Sn0.1@NPSC exhibits a uniform nanoparticle morphology, with diameters ranging from 15 to 75 nm (Figure 1g; Figure S1, Supporting Information). Figure 1h reveals the amorphous nature of the composite through high‐resolution TEM (HRTEM), which clearly demonstrates the disordered SnP_x_/SnS_x_ phases embedded within the amorphous carbon matrix. The corresponding selected‐area electron diffraction (SAED) pattern, exhibiting a characteristic diffuse halo, further confirms the short‐range ordered amorphous structure of SnP_x_/SnS_x_. Elemental mapping (Figure 1i–n) shows a homogeneous distribution of Sn, N, P, and S within the carbon matrix of Sn0.1@NPSC.

**Figure 2:**
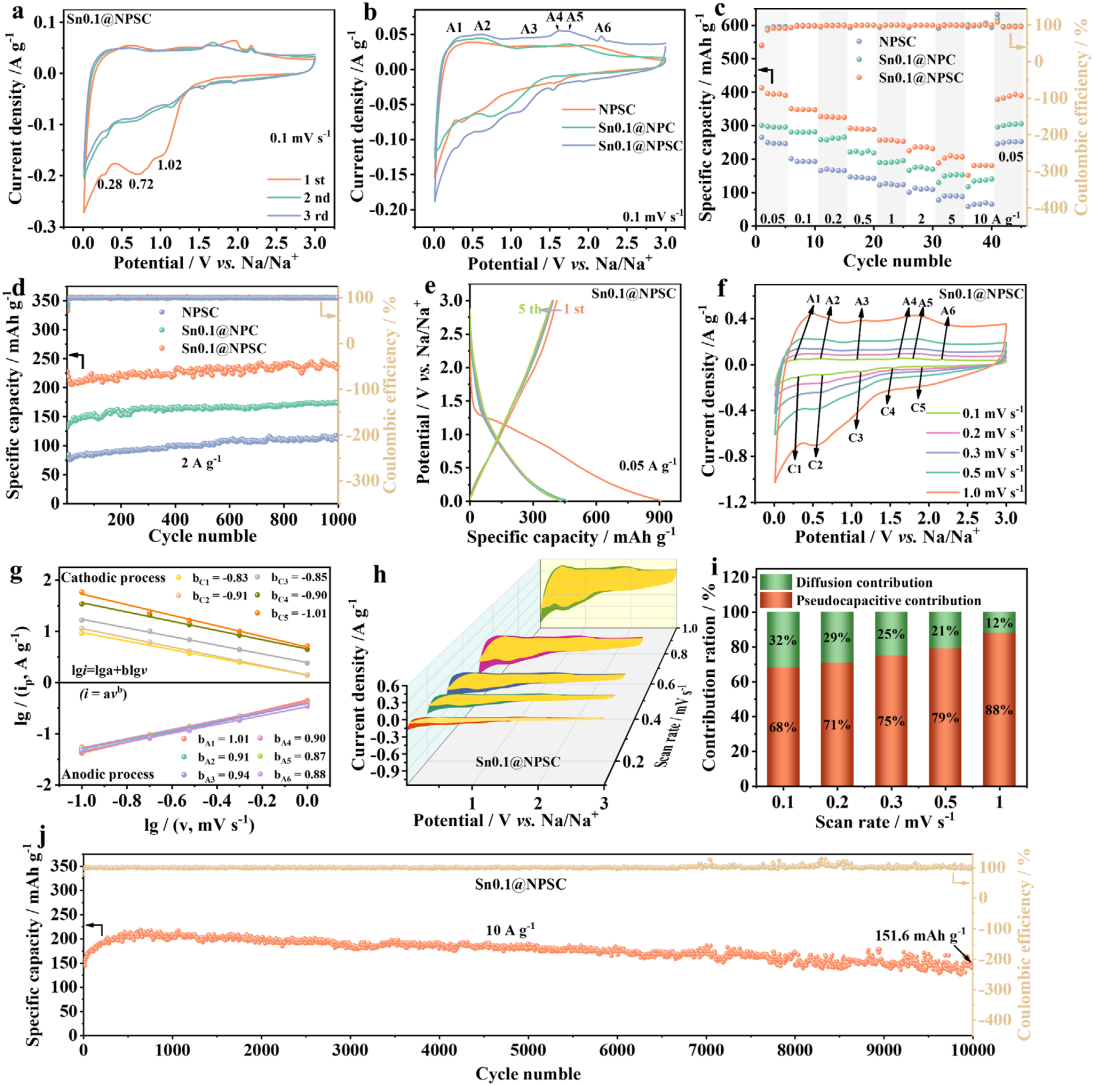
Electrochemical performance and kinetic analysis of Sn0.1@NPSC electrode. a) CV plot of the first three laps of Sn0.1@NPSC. b) CV plot at 0.1 mV s^−1^, c) rate performance, and d) cycling performance of the different electrodes. e) GCD plots for the first five laps, and f) CV plots at various scan rates, g) the relationship of lg*i* versus lg*v*, (h) pseudocapacitive contribution (yellow area), i) the values of pseudocapacitive contribution, and j) cycling performance of Sn0.1@NPSC electrode.

The sodium storage performance of three different electrode materials was evaluated in order to visualize the usefulness of introducing Sn and S in electrochemical performance. Three reduction peaks are observed during the first cathodic scan at ≈1.02, 0.72, and 0.28 V (**Figure** **2**a; Figure S2, Supporting Information). The broad peak near 1.02 V, which vanishes in subsequent cycles, is attributed to the formation of the solid electrolyte interface layer.^[^ ^41^ ^]^ The peak ≈0.72 V is associated with the reduction of SnP_x_/SnS_x_ by Na, leading to the formation of metallic Sn. The reduction peak at 0.28 V corresponds to the formation of the Na‐Sn alloy phase.^[^ ^42^ ^]^ The redox peaks in the second and third cycles overlap, indicating the high reversibility of these reactions. To identify the contributions of various redox peaks, Figure 2b shows the CV curves for electrodes containing different active elements (Sn, P, and S). During the anodic scan, peak A1, observed in NPSC, Sn0.1@NPC, and Sn0.1@NPSC, results from Na^+^ deintercalation from the graphite‐like carbon. Peak A2, observed in Sn0.1@NPC and Sn0.1@NPSC, is due to the desodiation of the Sn‐Na alloy. Peak A3, observed in NPSC, Sn0.1@NPC, and Sn0.1@NPSC, corresponds to the chemical desorption of sodium from the phosphorus‐functionalized carbon surface.^[^ ^43^ ^]^ Peaks A4 and A6 observed in Sn0.1@NPSC are related to the redox reactions of amorphous SnS_x_, likely involving a multi‐step conversion from Sn and Na_2_S to SnS_x_.^[^ ^44^ ^]^ Peak A5 observed in Sn0.1@NPC and Sn0.1@NPSC corresponds to the oxidation of irreversible reduction products from amorphous SnP_x_, likely due to Na_3_P desodiation to P.^[^ ^45^, ^46^ ^]^ These results suggest that Sn modifies the bonding environment of P and S within the slope‐dominant hard carbon matrix, thereby enhancing their sodium storage activity and contributing to increased overall capacity.

Figure 2c,d and Figure S3 (Supporting Information), illustrate the rate and cycling performance of the electrode materials. Sn0.1@NPSC exhibits the highest specific capacity of 412.8 mAh g⁻¹, outperforming Sn0.1@NPC (300.8 mAh g⁻¹) and NPSC (265.0 mAh g⁻¹). Even at an ultra‐high current density of 10 A g⁻¹, Sn0.1@NPSC retains a reversible capacity of 180.7 mAh g⁻¹, indicating excellent rate performance. As shown in Figure 2d, Sn0.1@NPSC retains the highest capacity of 228.5 mAh g⁻¹ after 1000 cycles. Both Sn0.1@NPSC and Sn0.1@NPC exhibit minimal capacity decay during cycling. This is attributed to the amorphous structure of SnP_x_/SnS_x_ and structural support provided by NPSC, which mitigates volume expansion during the conversion and alloying reactions. The gradual increase in capacity during cycling may be attributed to the dynamic activation process of the electrode materials: the NPSC mainly originates from the exposure of surface active sites and proliferation of pseudocapacitive sites in the carbon matrix, while the Sn0.1@NPC and Sn0.1@NPSC may involve both the surface activation of the carbon substrate and the optimization of the bulk‐phase alloying/conversion reaction of the active phase of the SnP_x_/SnS_x_. Moreover, it can also be seen from Figure S4 (Supporting Information) that Sn0.1@NPSC has the best content of Sn‐doped NPSC.

Figure 2e and Figure S5 (Supporting Information) show the first five GCD curves at 0.05 A g⁻¹ for Sn0.1@NPSC and other electrode materials. All three electrodes exhibit sloped GCD curves, characteristic of high‐rate electrode materials. To further explore the kinetic behavior of Sn0.1@NPSC, the kinetic b‐values for each redox peak are fitted (Figure 2f,g). Notably, the b‐values for all reduction and oxidation processes approach 1, indicating the pseudocapacitive‐dominant kinetic behavior of the Sn0.1@NSPC electrode. The contribution ratios of pseudocapacitive and diffusion‐controlled behavior are shown in Figure 2h,i, with pseudocapacitive contributions ranging from 68% to 88% at scan rates of 0.1–1.0 mV s⁻¹. The difference in the pseudocapacitive contribution arises from the varying proportions of surface‐controlled (capacitive‐type, NPSC) and diffusion‐controlled (Faradaic‐type, SnP_x_/SnS_x_) reactions within the material system, as well as the role of the components and structure in modulating both processes. Due to the ultrafast kinetics, Sn0.1@NSPC retains a stable reversible capacity of 151.6 mAh g⁻¹ at 10 A g⁻¹ after 10000 cycles (Figure 2j).

**Figure 3:**
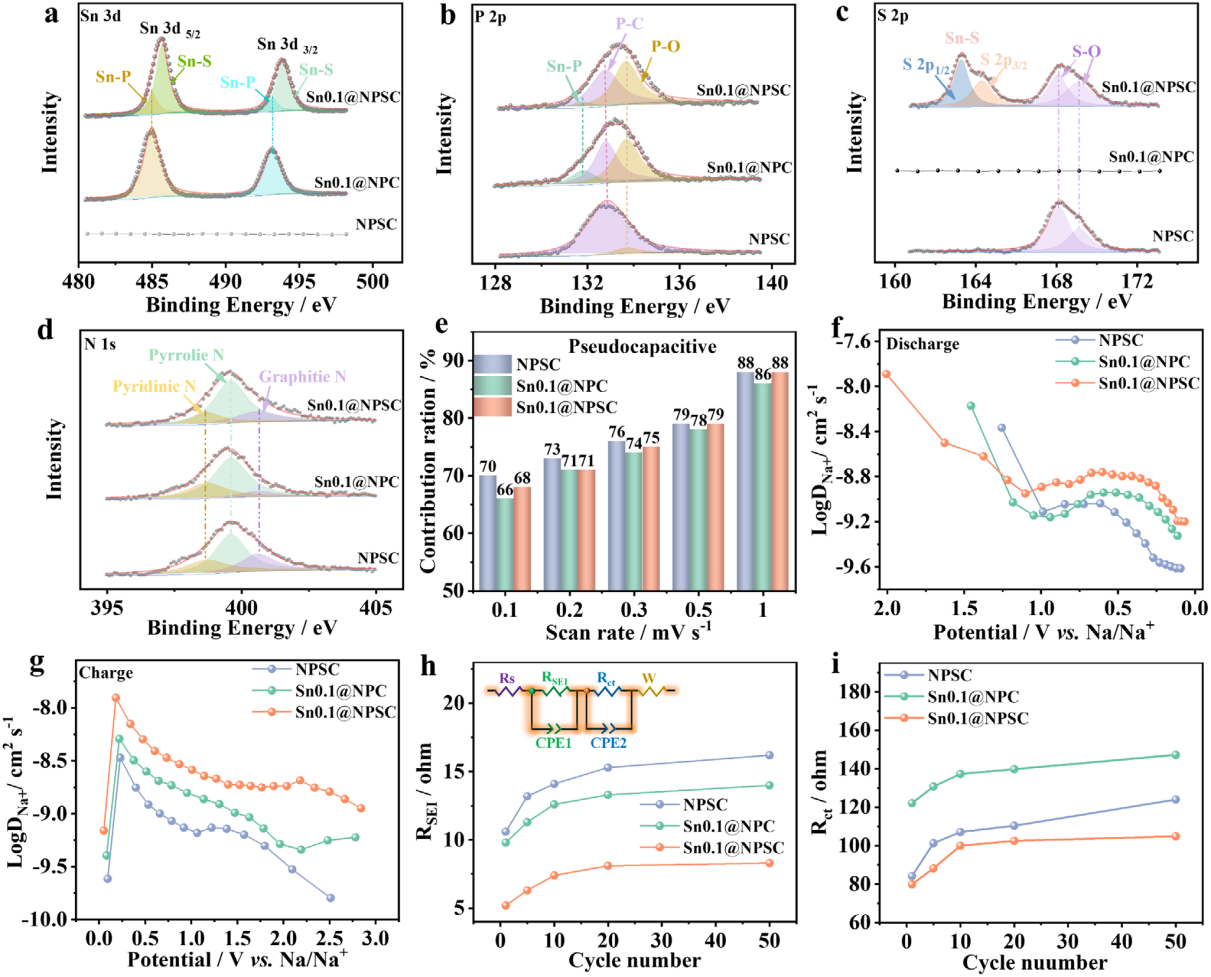
Structural, electrochemical, and kinetic characterization of NPSC, Sn0.1@NPC, and Sn0.1@NPSC electrodes. XPS data of a) Sn 3d, b) P 2p, c) S 2p, and d) N 1s spectra, e) pseudocapacitive contribution, Na^+^ diffusion coefficients during sodiation f) and desodiation g), the value of *R*_SEI_ h) and i) *R*_ct_ of the different electrode after different number of cycles.

To further assess the impact of Sn incorporation on structure and sodium ion storage behavior for the slope‐dominant hard carbon, a series of tests were performed. These tests compared the chemical composition, graphitization degree, pseudocapacitive contributions, Na⁺ diffusion coefficients, and charge transfer resistance across different electrodes. XPS analysis was used to probe the chemical composition and bonding states of the electrode surfaces, revealing the incorporation of Sn, P, and S into the slope‐dominant hard carbon. The introduction of Sn in NPSC leads to the appearance of characteristic Sn─S (468.6 eV) and Sn─P (467.3 eV) bonds in Sn0.1@NSPC (**Figure** **3**a). The binding energy positions of the Sn 3d₅/₂ and Sn 3d₃/₂ peaks in Sn0.1@NPC shift to a lower binding energy compared to Sn0.1@NSPC, further confirming the formation of the Sn─S bond.^[^ ^47^ ^]^ The P 2p XPS spectrum in Figure 3b shows that the Sn─P peak area in Sn0.1@NSPC is smaller than that in Sn0.1@NPC, suggesting that the introduction of S converts some Sn─P bonds into Sn─S bonds. Additionally, P─O and S─O bonds, originating from functional groups on the carbon surface, enhance the uniform distribution of Sn and provide additional active sites for pseudocapacitive behavior (Figure 3c).^[^ ^48^ ^]^ Heteroatom‐doped carbon materials, particularly nitrogen‐doped carbons, play a key role in tuning the electronic structure and surface chemistry.^[^ ^49^ ^]^ Edge nitrogen (pyridinic‐N, pyrrolic‐N) creates new active sites, while graphitic‐N improves the conductivity of the carbon framework.^[^ ^50^, ^51^ ^]^ The introduction of Sn reduces the proportion of graphitic‐N while increasing the content of edge nitrogen (Figure 3d). Meanwhile, S incorporation increases the graphitic‐N content, while reducing edge nitrogen, likely due to strong interactions between edge nitrogen and S.^[^ ^52^ ^]^ The relative changes in graphitic‐N content correlate with the changes in graphitization degree (A_D_/A_G_) shown in Figure S6 (Supporting Information). These findings suggest that Sn and S incorporation not only form new amorphous species but also modulate the graphitization degree and surface functional groups of the slope‐dominant hard carbon. The reducing N₂/10%H₂ atmosphere effectively inhibits both tin oxidation and nitrogen activation at 600 °C, thereby preventing the formation of SnO and SnN in Sn0.1@NPSC. XPS analyses confirm their absence, revealing no evidence of lattice oxygen (Sn─O) or metal‐nitrogen (Sn─N) bonding. Instead, oxygen exists solely as surface functional groups (C═O/O═C─OH/C─OH/C─O─C, Figure S7, Supporting Information), while nitrogen is present exclusively as dopant species (pyridinic/pyrrolic/graphitic N), further corroborating the lack of SnO and SnN phases in the material.

Figure 3e compares the pseudocapacitive contributions of different electrodes. Sn0.1@NSPC shows a pseudocapacitive contribution of 68% at 0.1 mV s^−1^, which is slightly lower than that of NPSC (70%) but higher than Sn0.1@NPC (66%). This suggests that while SnP_x_ enhances diffusion‐controlled behavior, the formation of SnS_x_ through S doping further promotes the pseudocapacitance contribution, improving the reaction kinetics. Sodium ion diffusion coefficients (*D*_Na⁺_) have been determined using the Galvanostatic Intermittent Titration Technique (GITT), as shown in Figure 3g,h and Figure S8 (Supporting Information). Sn0.1@NSPC exhibits the highest *D*_Na⁺_, outperforming other electrodes during both charging and discharging. The amorphous clusters and additional defects in Sn0.1@NSPC provide more sites for Na^+^ diffusion or adsorption, reducing diffusion resistance and thereby improving the diffusion coefficient. Electrochemical impedance spectroscopy (EIS) of the three electrodes at various cycle counts has been analyzed to obtain the changes in the interfacial resistance during cycling, as shown in Figure 3h,i and Figure S9 (Supporting Information). Both *R*_SEI_ and *R*_ct_ values of Sn0.1@NSPC are lower than those of NPSC, indicating that Sn incorporation reduces SEI resistance and enhances electron transfer at the electrode‐electrolyte interface. This reduction in interfacial resistance is likely attributed to the coupling of amorphous SnP_x_ and SnS_x_ with the carbon matrix (NPSC), which creates numerous heterogeneous interfaces and generates built‐in electric fields, thereby enhancing rapid electron transfer.

**Figure 4:**
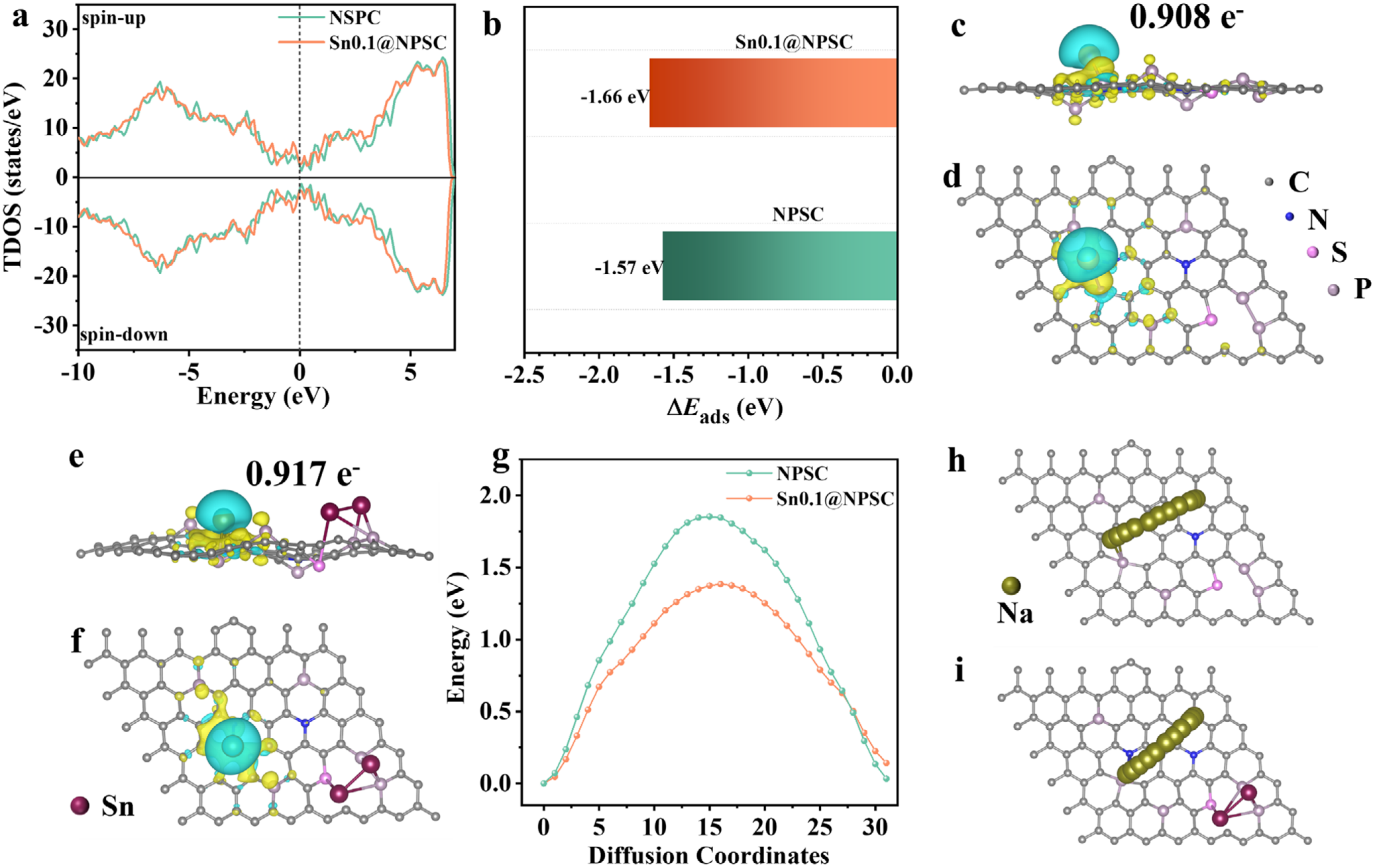
DFT calculation of NPSC and Sn0.1@NPSC. a) DOS, b) Na^+^ adsorption energy of NC700 of the NSPC and Sn0.1@NSPC. Bader charge analysis of the c,d) NSPC and e,f) Sn0.1@NSPC. g) The energy barrier for Na diffusion pathway on h) NPSC and i) Sn0.1@NSPC.

To further investigate theoretically the effect of Sn on the electronic properties and sodium storage behavior, density functional theory (DFT) calculations have been performed. As shown in the density of states (DOS) results in **Figure** **4**a, Sn0.1@NSPC exhibits a higher total density of states (TDOS) near the Fermi level compared to NSPC, indicating increased electron density and enhanced electronic conductivity due to the introduction of Sn.^[^ ^53^ ^]^ Comparison of sodium ion adsorption energies between Sn0.1@NSPC and NSPC reveals that Sn0.1@NSPC has a stronger adsorption energy, suggesting that the carbon heterostructure is more favorable for sodium ion adsorption (Figure 4b). Bader charge calculations were performed to analyze electron transfer. Electrons accumulate (yellow area) around N and P atoms, while sodium ions lose electrons (blue area), indicating electron transfer from sodium to N and P on the carbon substrate. Sn0.1@NSPC transfers 0.917 e⁻, which is higher than the 0.908 e⁻ transferred in NSPC, demonstrating superior charge transfer capabilities (Figure 4c–f). Furthermore, calculations of sodium ion diffusion energy barriers show that the diffusion energy barrier in the Sn0.1@NSPC model is significantly lower than in NSPC, indicating superior sodium ion diffusion kinetics (Figure 4g–i).

**Figure 5:**
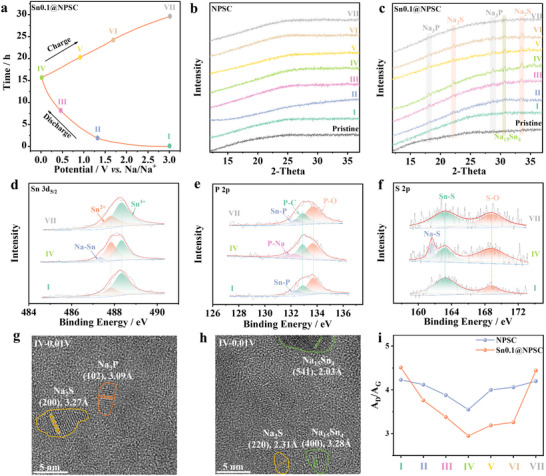
Energy storage mechanism investigation of the Sn0.1@NPSC electrode. a) GCD plots of the second cycle. Ex situ XRD of pristine electrode and the electrode at different potentials (I–VII) of the b) NPSC and c) Sn0.1@NPSC. Ex situ XPS spectrum of d) Sn 3d_5/2_, e) P 2p, f) S 2p for Sn0.1@NPSC electrode at different states. g,h) HRTEM of Sn0.1@NPSC discharged to 0.01 V. i) The corresponding A_D_/A_G_ values from ex situ Raman spectra of the NPSC and Sn0.1@NPSC electrodes at different potentials.

The sodium storage mechanism and the role of Sn in Sn0.1@NSPC have been investigated using ex situ XRD, ex situ XPS, ex situ HRTEM, and ex situ Raman. In order to better reflect the steady‐state electrochemical behavior of the materials, the reaction mechanism of the electrode materials during the second cycle of charging and discharging was investigated. **Figures** **5**a and S10 (Supporting Information) present the charge–discharge curves of Sn0.1@NSPC and NSPC during the second cycle and different potential points selected for subsequent electrode testing. As shown in Figure 5b, NPSC does not exhibit any significant additional characteristic peaks during cycling, indicating that P and S are predominantly present as surface functional groups on the carbon material. In Figure 5c, as sodiation progresses (I–IV), products related to SnP_x_ and SnS_x_, including Na_3_P (18.0° and 28.7°), Na_2_S (22.2° and 33.6°), and Na_15_Sn_4_ (30.7°), are formed. During desodiation (IV–VII), the intensities of the characteristic peaks for Na_3_P, Na_2_S, and Na_15_Sn_4_ gradually decrease or disappear.

To further elucidate the potential products from the conversion of amorphous SnP_x_/SnS_x_, ex situ XPS was employed. As seen in Figure 5d, the Sn 3d_5/2_ peak in the discharged state exhibits a blue shift compared to the charged state, suggesting the formation of Na_15_Sn_4_. Additionally, Figure 5e,f reveal the formation of Na_3_P and Na_2_S during discharging, indicating that the introduction of Sn activates the sodium storage capacity of P and S. HRTEM analysis of Sn0.1@NPSC discharged to 0.01 V reveals distinct lattice fringes indexed to the (102) planes of Na₃P, (200) and (220) planes of Na₂S, and (400) and (541) planes of Na₁₅Sn₄, demonstrating the phase transformation of sodiated products during deep discharge (Figure 5g,h).

The effect of Sn incorporation on the Na^+^ storage behavior of NPSC was further explored using ex situ Raman spectroscopy, as shown in Figure 5i and Figure S11 (Supporting Information). During discharge (I–IV), the gradual decrease in the A_D_/A_G_ in both NSPC and Sn0.1@NSPC reflects the adsorption of Na^+^ at defect sites. Sn0.1@NSPC exhibits a more significant variation in A_D_/A_G_ ratio during cycling, likely due to the presence of amorphous SnP_x_/SnS_x_, which promotes greater Na^+^ adsorption at defects in the hard carbon. Moreover, the A_D_/A_G_ variation during charge and discharge cycles of Sn0.1@NSPC and NSPC is nearly opposite, indicating highly reversible sodium storage behavior.^[^ ^54^ ^]^

**Figure 6:**
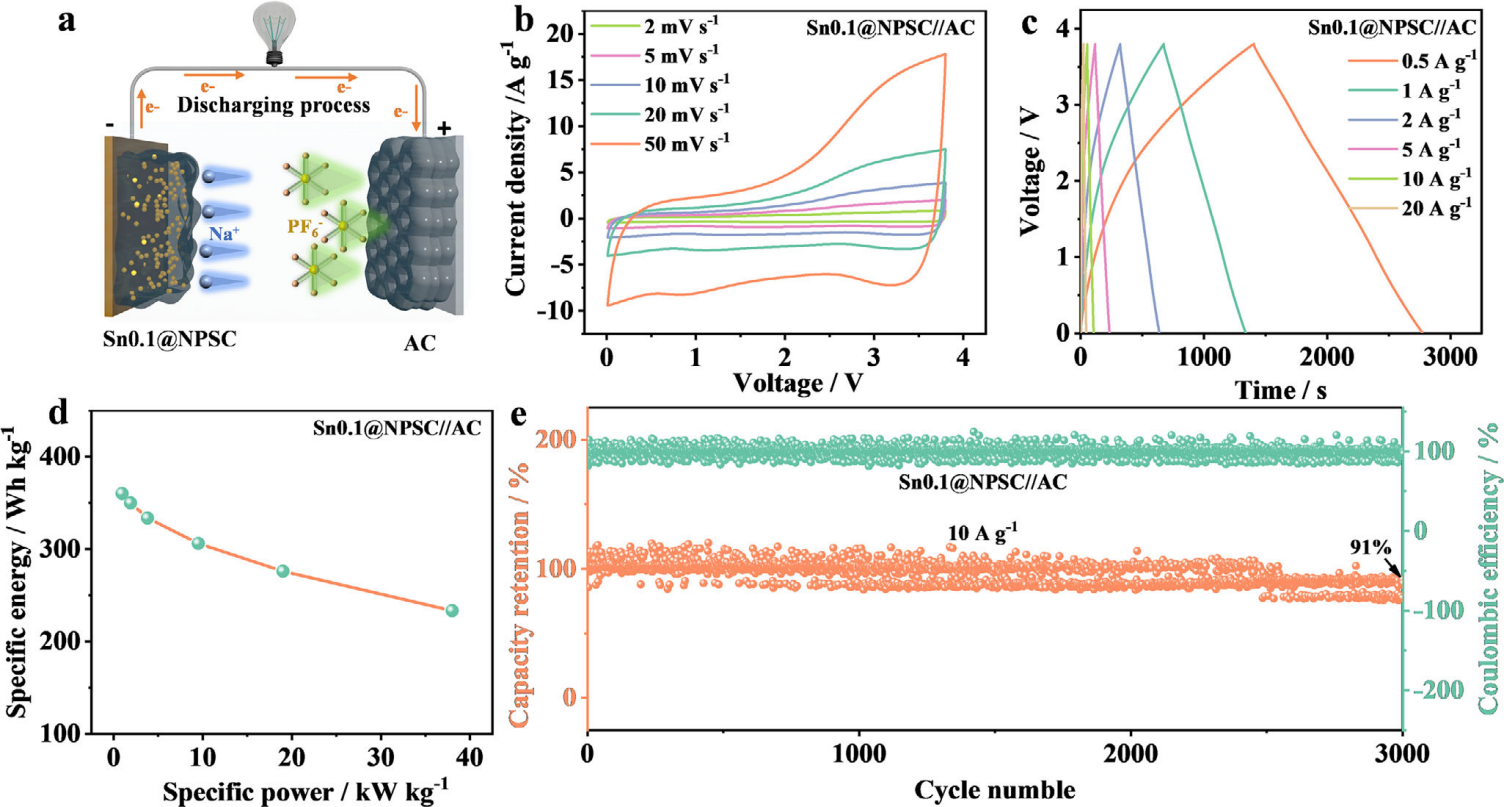
Electrochemical performance and working mechanism of Sn0.1@NPSC//AC SIC. a) The working schematic, b) CV plots, c) GCD curves, d) Ragone plots and e) cycling performance.

To evaluate the feasibility of Sn0.1@NSPC for SIC, a device was assembled by pairing it with an activated carbon (AC) cathode. **Figure** **6**a illustrates the working principle of the SIC, which involves sodiation–desodiation of the Sn0.1@NSPC anode and adsorption–desorption of PF_6_^−^ on the AC cathode during charge–discharge cycles. As shown in Figure 6b, the working voltage window is optimized to 0.01–3.8 V to minimize side reactions. The CV curves of the SIC show minimal deviation from an ideal rectangular shape, reflecting the combined capacitive and battery‐type energy storage mechanisms of both the anode and cathode. Figure 6c presents GCD curves at 0.5–20A g^−1^, which exhibit quasi‐triangular shapes, further confirming the coexistence of capacitive and battery‐type storage mechanisms. From the charge–discharge curves, the Ragone plot of the Sn0.1@NSPC//AC SIC based on the anode mass (Figure 5d) demonstrates an energy density of 360 Wh kg^−1^ at a power density of 950 W kg^−1^. Notably, even at an ultra‐high power density of 38 kW kg^−1^, the energy density remains 223 Wh kg^−1^, indicating a higher power density compared to recent reports in the literature (Table S1, Supporting Information). Importantly, Sn0.1@NSPC//AC SIC also exhibits outstanding cycling stability (Figure 6e), with 91% retention of energy density after 3000 cycles. These results highlight the promising potential of Sn0.1@NSPC as an advanced anode material for SIC.

## 3 Conclusion

To address the critical challenge of kinetic and cycling life mismatch in sodium‐ion hybrid capacitors, this study designed and successfully fabricated a novel composite anode (Sn0.1@NPSC) with slope‐dominant charge–discharge characteristics, achieved through the confinement of amorphous SnP_x_/SnS_x_ phases within an N/P/S‐co‐doped hard carbon matrix. The isotropic structure of amorphous SnP_x_/SnS_x_ synergizes with interfacial Sn─P/Sn─S chemical bonding to suppress volumetric expansion during cycling. DFT calculations reveal that Sn doping reduces sodium‐ion diffusion barriers and improves electronic conductivity, contributing to ultra‐long cycling stability (90% capacity retention after 10 000 cycles). The Sn‐doped hard carbon framework constructs a 3D conductive network through optimized mesoporous architecture and reduced charge transfer resistance, while activating multi‐step redox reactions (reversible Na₃P/Na₂S formation) and pseudocapacitance‐dominated kinetics (88% contribution at 1 mV s⁻¹). These structural advantages collectively enable a high specific capacity of 412.8 mAh g⁻¹ at 0.05 A g⁻¹ and exceptional rate capability (180.7 mAh g⁻¹ at 10 A g⁻¹). Sn0.1@NSPC//AC SIC evaluations demonstrate practical applicability with an energy density of 360 Wh kg⁻¹ (anode‐based) and 91% capacity retention over 3000 cycles. By integrating multiscale characterization and theoretical analysis, this work offers valuable insights into how amorphous alloy‐carbon interfaces facilitate strain accommodation and charge transport, thereby providing new directions for designing high‐performance sodium storage materials and broadening the application scope of amorphous alloy–carbon systems.

## 4 Experimental Section

### Preparation of NPSC, Sn0.1@NPC, Sn0.1@NPSC Anode Materials

To prepare Sn0.1@NPSC, 0.35 g of SnCl₄·4H₂O was dissolved in 80 mL of deionized water and stirred for 0.5 h. Next, 600 µL of aniline, 3 mL of phytic acid (70% in H_2_O), and 20 mL of an aqueous solution containing 1.5 g of ammonium persulfate were added sequentially. The mixture was stirred at 1.5 °C for 12 h, followed by freeze‐drying. The resulting material was washed thoroughly with deionized water and ethanol, then vacuum‐dried to obtain Sn0.1@PANI (Sn‐doped polyaniline). The obtained Sn0.1@PANI was ground manually with sulfur (Sn0.1@PANI: S = 1:2 by mass) using a mortar and pestle. This mixture was pyrolyzed at 600 °C for 4 h under a N_2_ atmosphere containing 10% H₂. After cooling to room temperature, the final product, Sn0.1@NPSC, was collected. Sn0.1@NPC was synthesized using the same procedure as Sn0.1@NPSC, except that sulfur was omitted during the pyrolysis of Sn0.1@PANI. Sn0.05@NPSC and Sn0.2@NPSC were prepared using identical protocols to Sn0.1@NPSC, but with SnCl₄·4H₂O precursor quantities varied to 0.175 and 0.7 g, respectively, for controlled Sn doping levels. Similarly, NPSC was prepared following the same protocol as Sn0.1@NPSC, except that SnCl₄·4H₂O was excluded during the PANI synthesis step.

### Characterization Measurements

The synthesized powders and electrode were characterized using X‐ray diffraction (XRD, SmarLab3KW) to analyze their phases. The microstructure of the samples was examined with scanning electron microscopy (SEM, Phenom proX) and transmission electron microscopy (TEM, JEOL JEM‐F200). The Brunauer–Emmett–Teller (BET) method, combined with Barrett–Joyner–Halenda (BJH) analysis of nitrogen adsorption–desorption isotherms (Autosorb‐IQ3), was employed to measure specific surface area (SSA) and pore‐size distribution. The surface chemical composition and electronic structure of the powders and electrode were identified via ex situ X‐ray photoelectron spectroscopy (XPS, Thermo Scientific K‐Alpha). Thermogravimetric analysis (TG, STA 449 F5) and inductively coupled plasma optical emission spectrometry (ICP‐OES, Thermo Fisher iCAP PRO) were used to determine the element content. Fourier‐transform infrared (FTIR, ALPHA) spectroscopy and Raman spectroscopy (Horiba LabRAM HR Evolution) were conducted to investigate the surface and chemical structures of the samples.

### Electrochemical Measurements

All anode electrodes (Sn0.1@NPSC, Sn0.1@NPC, and NPSC) were prepared by mixing 70 wt.% active material, 20 wt.% conductive carbon black, and 10 wt.% polyacrylic acid in N‐methylpyrrolidone (NMP). The resulting slurry was coated onto copper foil and dried in a vacuum oven at 80 °C for 12 h. The mass loading of the anode active material was controlled to be ≈0.8 mg cm⁻^2^. For half‐cell measurements, metallic sodium was used as both the counter electrode and the reference electrode. The cathode electrodes (activated carbon, AC, XFNANO, XFP06) were fabricated similarly by mixing 70 wt.% active material, 20 wt.% conductive carbon black, and 10 wt.% polyvinylidene fluoride in NMP. The slurry was coated onto aluminum foil and dried in a vacuum oven at 80 °C for 12 h. The Sn0.1@NPSC anode was pre‐activated in a half‐cell at 0.1 A g⁻¹ for five cycles before assembling the SIC. Under optimized mass loading conditions (cathode: anode = 4:1), SICs were constructed using Sn0.1@NPSC as the anode and AC as the cathode. The CR2025 coin cells were assembled in an argon‐filled glovebox. Glass fiber (GF/A) served as the separator. The electrolyte consisted of 1.0 m NaPF₆ dissolved in a mixture of ethylene carbonate, dimethyl carbonate, and ethyl methyl carbonate (volume ratio 1:1:1), with the addition of 5 wt.% fluoroethylene carbonate as an additive. The cycling and rate performances of the cells were evaluated using a battery testing system (LAND CT2001A, Wuhan, China). Cyclic voltammetry (CV) and electrochemical impedance spectroscopy (EIS, 10⁻^1^–10⁵ Hz) were performed using an electrochemical workstation (Chenhua, CHI660e).

### DFT Simulations

All spin‐polarized DFT calculations were carried out utilizing the projector augmented wave framework to describe the interactions between electrons and ions, implemented within the VASP software. The electron exchange‐correlation effects were treated using the Perdew–Burke–Ernzerhof functional within the generalized gradient approximation. To account for long‐range dispersion forces, the Grimme DFT‐D3 scheme was employed, which was crucial for accurately representing van der Waals interactions. The energy cutoff for the plane‐wave basis set was configured at 400 eV, and Brillouin zone sampling was performed using a gamma‐centered 3 × 3 × 1 k‐point grid. The convergence criteria for electronic self‐consistency were set to 10^−5^ eV, while the force convergence threshold was established at 0.02 eV Å^−1^. Additionally, a vacuum layer of 15 Å was included perpendicular to the interface to minimize interactions with periodic images.

## Conflict of Interest

All authors declared that there are no conflicts of interest.

## Supporting information



Supporting Information

## Data Availability

The data that support the findings of this study are available from the corresponding author upon reasonable request.
