# Supplementary material for: Active Component Design of Amorphous SnPx/SnSx and Interfacial Bonding Engineering in N/P/S‐Doped Hard Carbon for High‐Rate Sodium‐Ion Hybrid Capacitors
Source: Adv Sci (Weinh). 2025 May 28;12(32):e06532. doi: 10.1002/advs.202506532 (PMC12407265; doi:10.1002/advs.202506532)
Supplement: Supplementary file 1 — Supporting Information [file ADVS-12-e06532-s001.docx]

**Supporting information (SI):**

**Active Component Design of Amorphous SnPₓ/SnSₓ and Interfacial Bonding Engineering in N/P/S-Doped Hard Carbon for High-Rate Sodium-Ion Hybrid Capacitors**

Ziyang Jia^1^, Yichen Duan ^1^, Xi Chen^1^, Zewen Sun ^1^, Lili Liu^1,^*, Lijun Fu ^1^, Yuhui Chen ^1^, Faxing Wang ^2^, Tao Wang ^2,*^, Yuping Wu^1,2*^

^1^ School of Energy Science and Engineering, Nanjing Tech University, 211816 Nanjing, Jiangsu Province, China.

^2^ Confucius Energy Storage Lab, School of Energy and Environment & Z Energy Storage Center, Southeast University, Nanjing 211189, China.


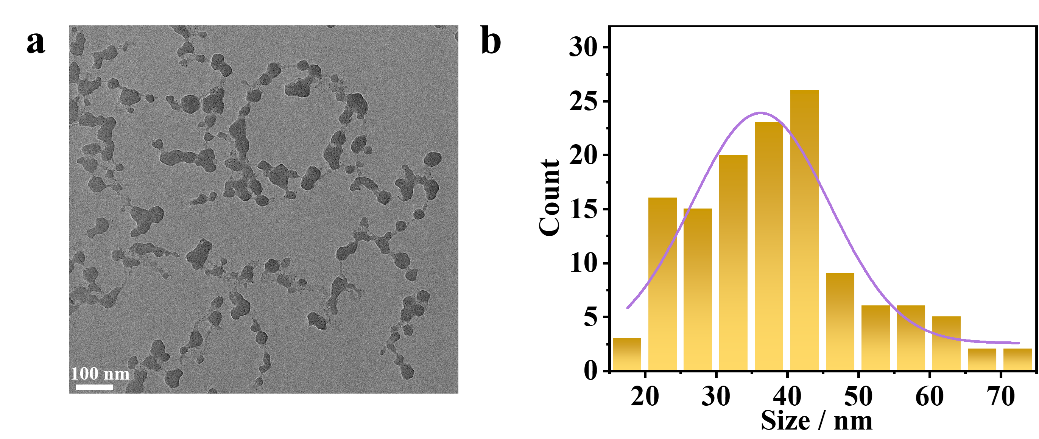


**Figure. S1** (a) TEM and (b) particle size distribution of Sn0.1@NPSC.


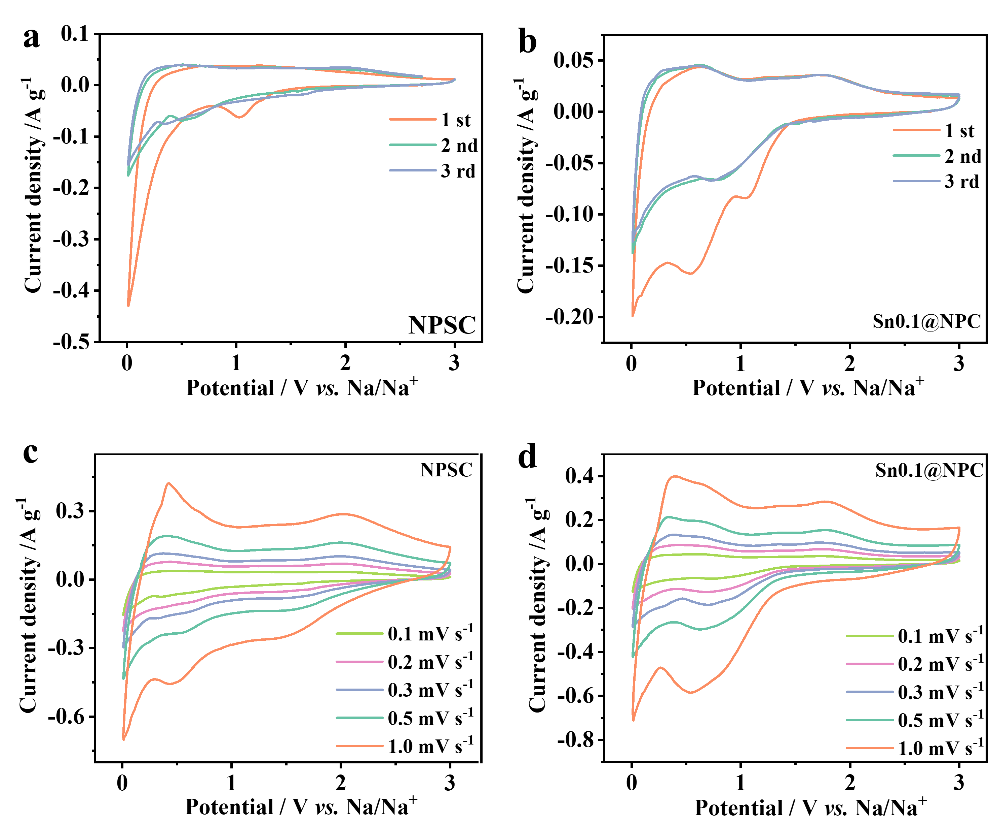


**Figure. S2** The first three laps CV plots at 0.1-1 mV s^-1^ for (a) NPSC and (b) Sn0.1@NPC. The CV plots at 0.1-1 mV s^-1^ for (c) NPSC and (d) Sn0.1@NPC.


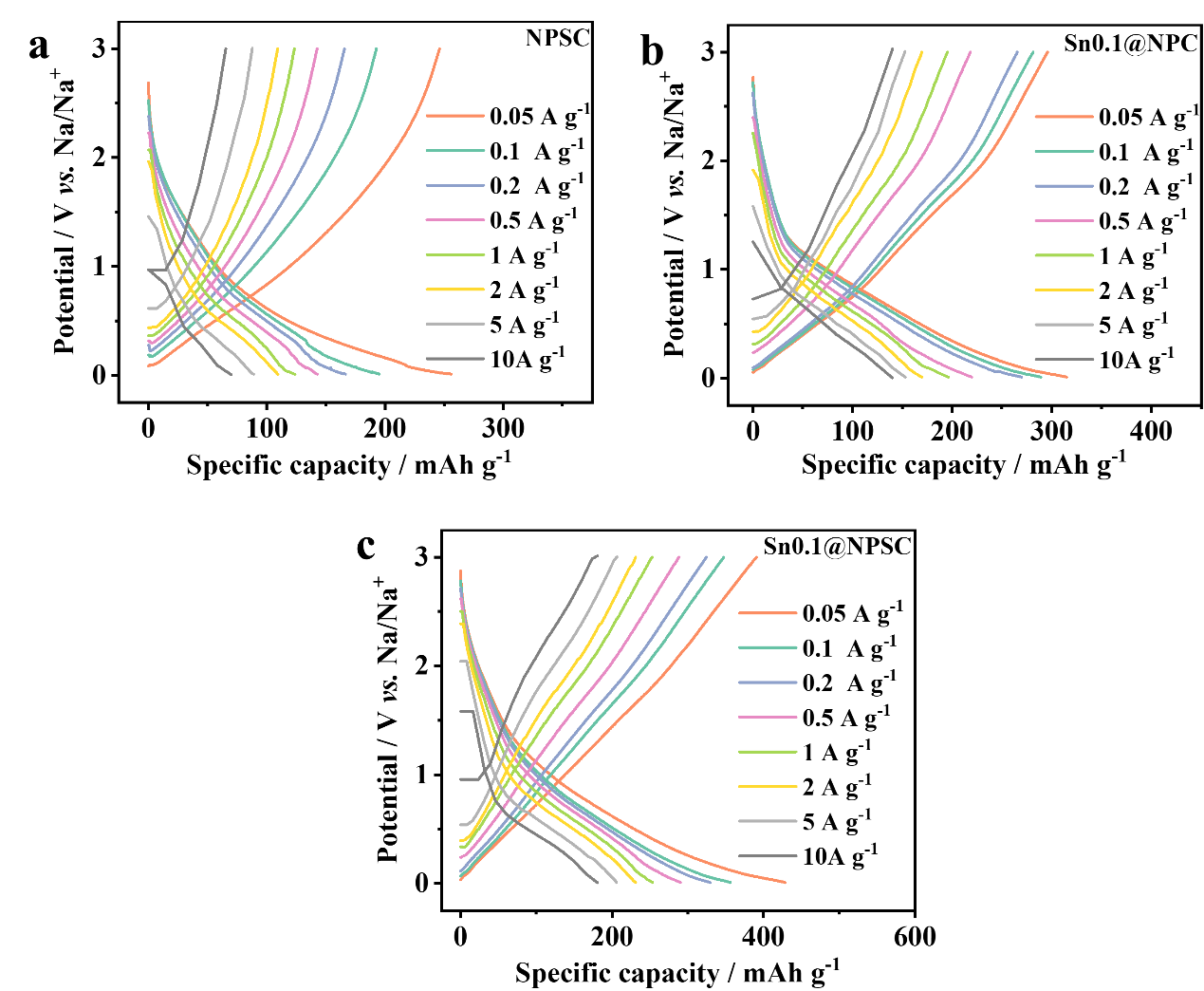


**Figure. S3** The GCD curves at 0.05-10 A g^-1^ for (a) NPSC, (b) Sn0.1@NPC.and (c) Sn0.1@NPSC.


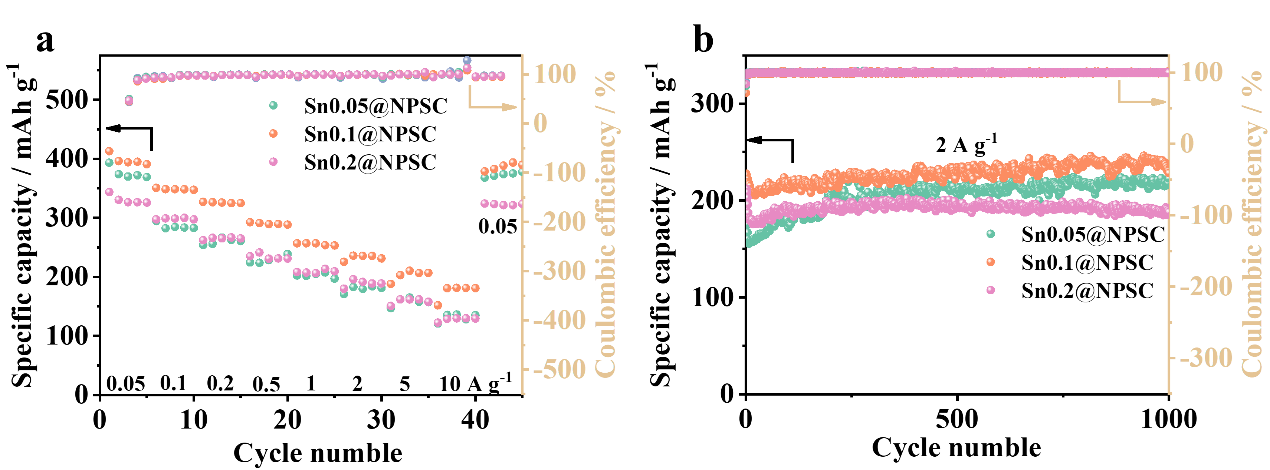


**Figure. S4** (a) rate performance and (b) cycling performance of the Sn0.05@NPSC, Sn0.1@NPSC and Sn0.2@NPSC electrode.


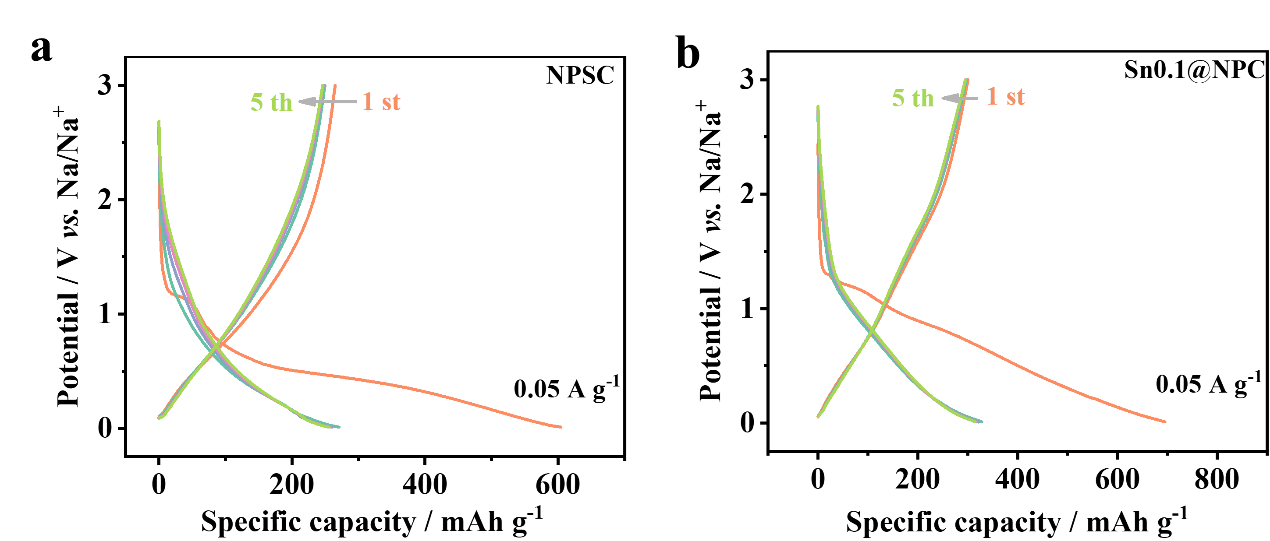


**Figure. S5** The first five laps GCD curves at 0.05 A g^-1^ for (a) NPSC and (b) Sn0.1@NPC.


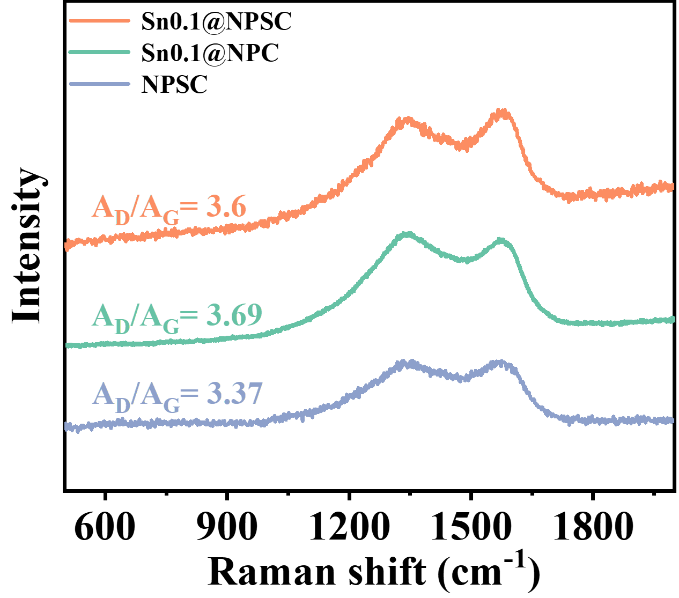


**Figure. S6** Raman of NPSC, Sn0.1@NPC and Sn0.1@NPSC.


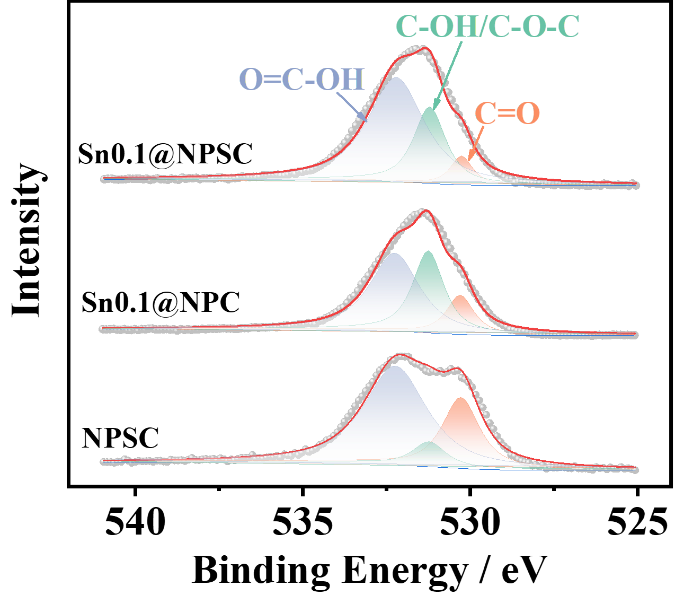


**Figure. S7** O1s of NPSC, Sn0.1@NPC and Sn0.1@NPSC.


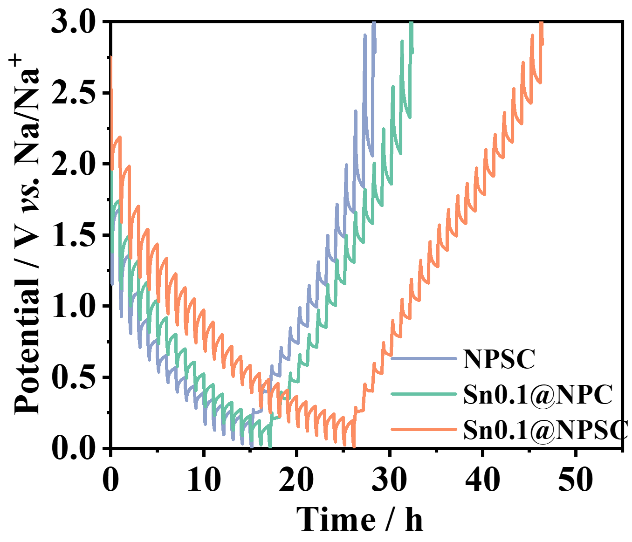


**Figure. S8** GITT curves of NPSC, Sn0.1@NPC and Sn0.1@NPSC electrode.


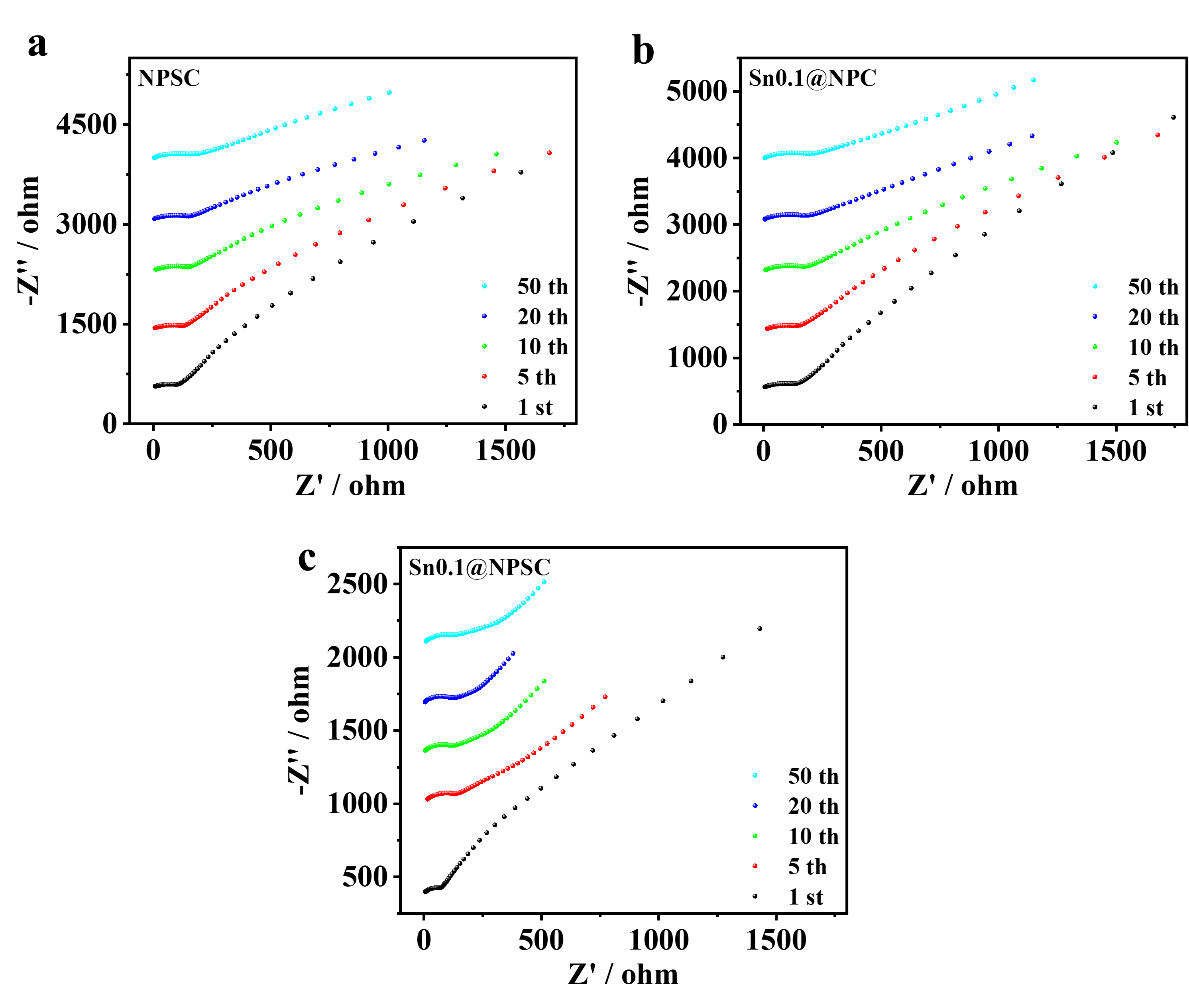


**Figure. S9** Nyquist plots after different number of cycles for (a) NPSC, (b) Sn0.1@NPC and (c) Sn0.1@NPSC electrode.


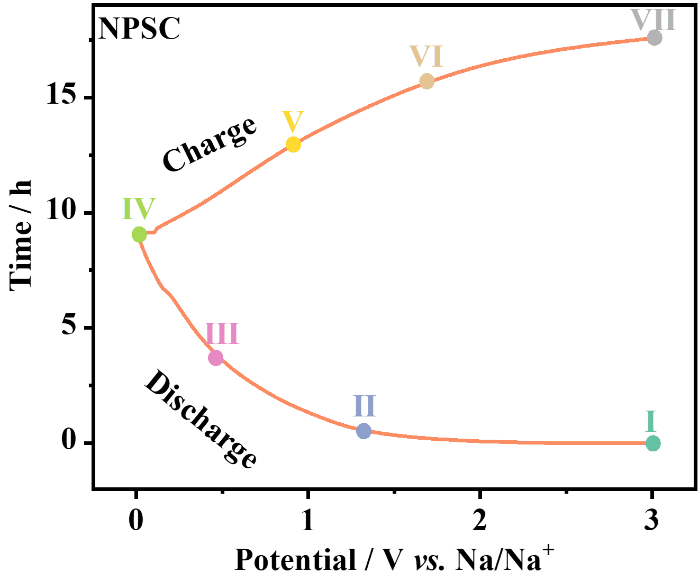


**Figure. S10** The second GCD curves at 0.1 A g^-1^ for NPSC.


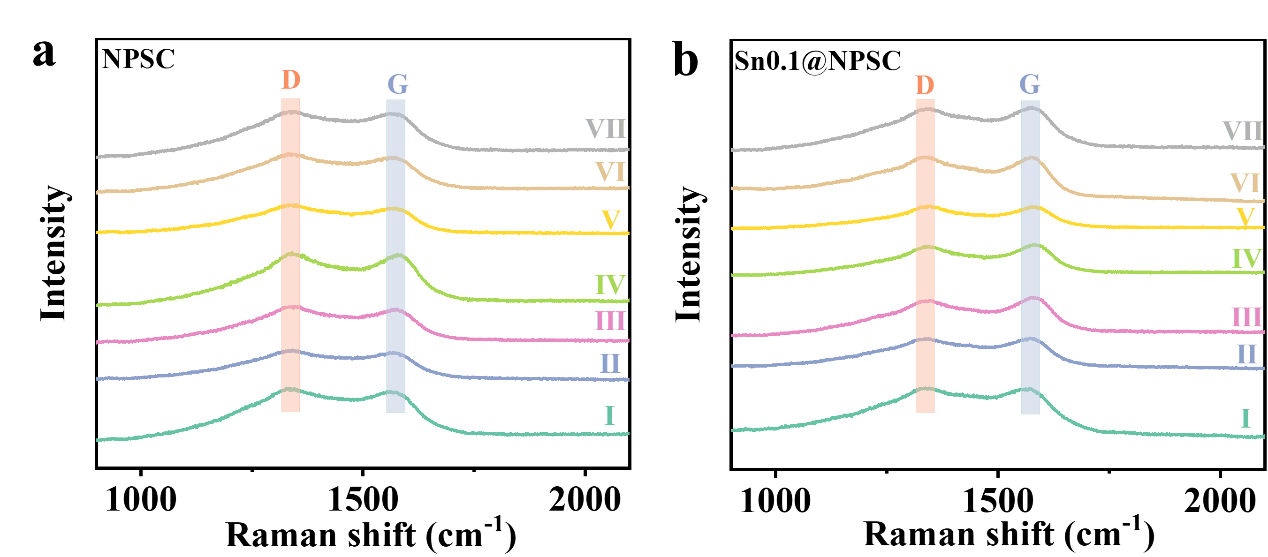


**Figure. S11** Ex-situ Raman of (g) NPSC and (h) Sn0.1@NPSC electrode at different potential.

**Table S1.** A comparison for the performance of the Sn0.1@NPSC//AC in this work with some representatively reported SICs (The energy/power densities of all devices are calculated based on the mass of the anode).

| SIC devices | Energy density  (Wh/kg) | Power density  (W/kg) | Capacitance retention/ cycles | Ref. |
| --- | --- | --- | --- | --- |
| MSC//AC | 327-147 | 294-15408 | 88%/2000 | [1] |
| VSSe/C//AC | 202.8 | 15200 | 96.6%/2000 | [2] |
| KNZMF@rGO//AC | 128.8-112 | 1120-22400 | 51.5%/1000 | [3] |
| MoS_2_@Ti_3_C_2_T_x_//AC | 286.2 | 266.6 | 81.1%/15000 | [4] |
| MoO_2_ SCs@N-CNFs//AC | 284.8-133.2 | 300-12000 | 60%/2000 | [5] |
| TA-DH-COF/GA//AC | 316.5-168 | 300-30000 | 88.8%18000 | [6] |
| FS/TO@C//AC | 368.5-103 | 500-50000 | 74.3%/1000 | [7] |
| PCC//HCC | 369-264 | 357-36456 | 92%/1000 | [8] |
| **Sn0.1@NPSC//AC** | **360-223** | **950-38000** | **91%/3000** | **This work** |

**References**

1. H. Y. Zhang, B. L. Liu, Z. J. Lu, J. D. Hu, J. Xie, A. Z. Hao, Y. L. Cao, *Small* **2023**, 19, 2207214.

2. J. Wang, J. Cui, Z. J. Li, D. Zhang, H. L. Sun, H. Wang, Q. J. Wang, H. J. Woo, S. Ramesh, B. Wang, *Chem. Eng. J.* **2023**, 464, 142764.

3. F. Yang, R. Ding, Z. Y. Jia, W. J. Yu, Y. Li, A. L. Wang, M. Liu, J. M. Xie, M. Yan, Q. Fang, Y. Z. Zhang, X. J. Sun, E. H. Liu, *Energy Storage Mater.* **2022**, 53, 222.

4. Y. H. Xiao, Q. L. Le, Y. Kong, W. D. Lv, D. C. Su, X. H. Rui, S. Y. Yu, A. Q. Zhang, J. H. Zhao, Q. X. Yang, Q. X. Jin, Y. J. Zheng, Y. Yu, S. M. Fang, *Chem. Eng. J.* **2025**, 505, 159268.

5. Y. Liu, S. C. Wang, X. Sun, J. Y. Zhang, F. uz Zaman, L. R. Hou, C. Z. Yuan, *Energy Environ. Mater.* **2023**, 6, e12263.

6. W. Y. Yuan, J. Y. Weng, Y. R. Sun, P. J. Zhang, M. H. Ding, S. Y. Chen, P. F. Zhou, J. Zhou, *Adv. Funct. Mater.* **2025**, 35, 2415402.

7. X. H. Xiao, X. G. Duan, Z. R. Song, X. L. Deng, W. T. Deng, H. S. Hou, R. J. Zheng, G. Q. Zou, X. B. Ji, *Adv. Funct. Mater.* **2022**, 32, 2110476.

8. Y. Y. Wang, Z. Y. Wang, Y. J. Xu, W. H. Chen, G. S. Shao, B. H. Hou, *Energy Environ. Sci.* **2024**, 17, 6811.
